# Association of IL-6, IL-8, MMP-13 gene polymorphisms with knee osteoarthritis susceptibility in the Chinese Han population

**DOI:** 10.1042/BSR20181346

**Published:** 2019-02-01

**Authors:** Gang Sun, Cheng-Lei Ba, Ren Gao, Wenqing Liu, Qiang Ji

**Affiliations:** Department of Spinal Surgery, Qingdao Central Hospital, Qingdao 266042, China

**Keywords:** Interleukin-6, Interleukin-8, Matrix metalloproteinase-13, Osteoarthritis, Single nucleotide polymorphism

## Abstract

**Objective:** To identify the association between the interleukin (IL) 6 (*IL-6*) rs1800795 (-174 G>C), *IL-8* rs4073 (-251T>A), and matrix metalloproteinase-13 (*MMP-13*) rs2252070 (-77G>A) gene polymorphisms and knee osteoarthritis (KOA) susceptibility in the Chinese Han population. **Methods:** Genomic DNA was extracted from a total of 400 KOA patients and 400 healthy subjects. Sanger sequencing was performed to determine the genotypes of the *IL-6* rs1800795 (-174 G/C), *IL-8* rs4073 (-251A/T), and *MMP-13* rs2252070 (-77A/G) loci. The mRNA expression levels of *IL-6, IL-8*, and *MMP-13* in osteoblasts and the protein expression levels of IL-6, IL-8, and MMP-13 in the synovial fluids of KOA patients were analyzed. **Results:** The recessive model of IL-6 rs1800795 locus was found to be associated with KOA risk (adjusted odds ratio (OR) = 1.657, 95% confidence interval (CI) = 1.396–1.866, *P*<0.001). The *IL-8* rs4073 locus dominant and recessive model showed no significant association with KOA risk (*P*>0.05). The dominant and recessive models of the *MMP-13* rs2252070 locus showed higher risk for developing KOA (dominant model: adjusted OR = 1.271, 95%CI = 1.095–1.480, *P*=0.001; recessive model: adjusted OR = 1.361 95%CI = 1.151–1.569, *P*<0.001). The G>C mutation in IL-6 rs1800795 and the G>A mutation in *MMP-13* rs2252070 were associated with significantly higher KOA disease severity. The G>C mutation in the IL-6 rs1800795 locus was associated with up-regulation of IL-6 expression. The G>A mutation in the *MMP-13* rs2252070 locus was associated with up-regulation of MMP-13 expression. **Conclusion:** The *IL-8* rs4073 (-251T>A) mutation was not associated with KOA susceptibility. The *IL-6* rs1800795 (-174 G>C) and *MMP-13* rs2252070 (-77G>A) mutations were associated with KOA susceptibility, increased disease severity, and up-regulation of IL-6 and MMP-13 expression levels.

Osteoarthritis (OA) is a chronic progressive osteoarthropathy that is characterized by degenerative changes in articular cartilage and hyperosteogeny [1]. In recent years, the development of OA in the Chinese population has been associated with ageing, and OA incidence has gradually increased. In particular, knee OA (KOA) showed the highest incidence and has become a major public health concern, particularly in elderly patients [2,3].

Recent studies have shown that the occurrence of OA is influenced by obesity, inflammation, ageing, injury, and genetic factors [4–6]. The degeneration and destruction of articular cartilage are the most important pathological changes that occur during OA. Biological and mechanical mechanisms play important roles in the progression of OA. Biological mechanisms are mediated by pro-inflammatory cytokines and proteases, which promote cartilage degradation and progression [7]. Current OA treatment is primarily focussed on articular cartilage, and these treatments often ignore the bone tissues surrounding the cartilage. Studies have shown that osteophyte formation is associated with narrowing of the joint space [8]. In addition, some studies indicated changes in gene expression patterns during cartilage degeneration in osteophytes in clinical cases. Studies have reported that non-physiological mechanical stress caused by joint deformities can up-regulate the expression of interleukin (IL) 6 (IL-6), IL-8, and matrix metalloproteinase-13 (MMP-13) in the callus and is potentially involved in OA progression [9]. Single nucleotide polymorphisms (SNPs) in the genes encoding *IL-6* [10], *IL-8* [11], and *MMP-13* [12] have been associated with the occurrence of various diseases. However, few studies have investigated the occurrence of OA. In the present case–control study, we analyzed the association of SNPs in the promoter regions of *IL-6* rs1800795 (-174G>C), IL-8 rs4073 (-251T>A), and rs2252070 (-77G>A) *MMP-13* with KOA susceptibility and disease severity. In addition, we analyzed the correlation of the rs1800795 (-174G>C) in the *IL-6* locus, rs4073 (-251T>A) in the *IL-8* promoter region, and rs2252070 (-77G>A) in the *MMP-13* promoter region with KOA susceptibility and disease severity.

## Materials and methods

### Patient characteristics

A total of 400 patients with KOA undergoing artificial joint replacement surgery at Zhejiang Provincial People’s Hospital from May 2014 to May 2017 were recruited in the case group. The case group comprised 184 males and 216 females who were 25–81 years old, with an average age of 56.6 ± 9.3. A total of 400 healthy subjects were recruited in the control group. The control group comprised 189 males and 211 females who were 27–80 years old, with an average age of 55.7 ± 10.5. KOA was diagnosed based on the KOA standards established by the Association of Rheumatology Health Professionals (ARHP) [13]. The following exclusion criteria were considered: joint diseases caused by other causes, such as inflammatory arthritis, septic arthritis, traumatic arthritis, chronic inflammation, infectious diseases, and tumor or skeletal dysplasia; diseases that can cause systemic inflammatory reactions; and failure to obtain KOA patients with joint effusion. The control group underwent clinical examination. X-ray analysis confirmed the absence of KOA or other types of arthritis and no evident symptoms of joint pain, including pain, swelling, tenderness, and limited mobility. The present study was approved by the Medical Ethics Committee of Zhejiang Provincial People’s Hospital. All participants provided written informed consent.

### Genotyping

**Figure 1:**
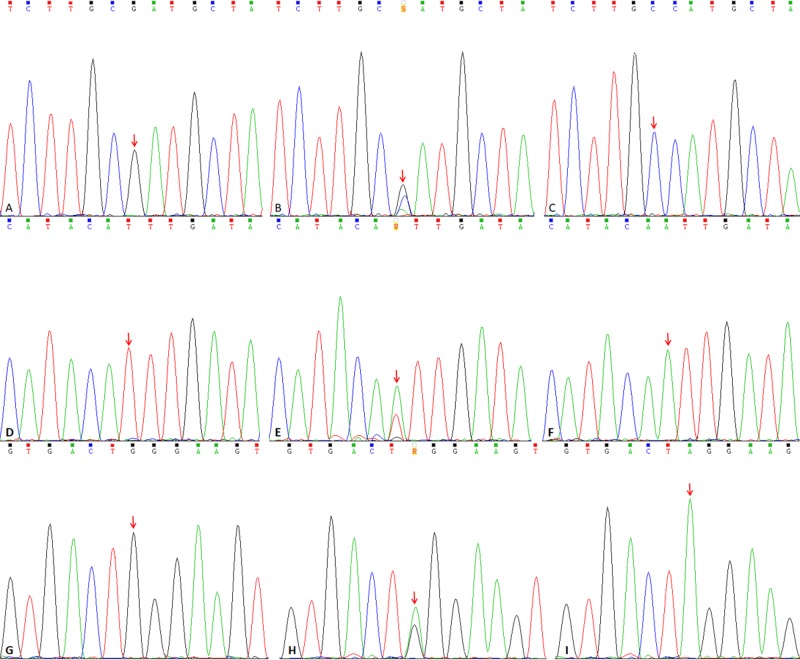
Sanger sequencing of target loci harboring the *IL-6* rs1800795, *IL-8* rs4073, and *MMP* rs2252070 polymorphisms (**A**–**C**) Sanger sequencing results for KOA patients harboring the GG, GC, and CC genotypes at the *IL-6* rs1800795 locus. (**D**–**F**) Sanger sequencing results for KOA patients harboring the TT, TA, and AA genotypes at the *IL-8* rs4073 locus. (**G**–**I**) Sanger sequencing results for KOA patients harboring the GG, GA, and AA genotypes at the *MMP-13* rs2252070 locus.

Venous blood sample (2 ml) was collected from each subject. Genomic DNA was extracted using the QIAamp DSP DNA Blood Mini Kit (Cat No. 61104, Qiagen, Germany) and subsequently stored at −80°C in a freezer. PCR primers were designed based on the *IL-6, IL-8*, and *MMP-13* sequences using Primer Premier 5.0 (Premier, Palo Alto, CA, U.S.A.) (Table 1). PCR amplification was performed in 20 μl reaction volumes containing 2.0 μl of 10× PCR buffer, 2.0 μl of 0.3 mmol/l dNTPs, 1.0 μl each of the upstream (10 μM) and downstream primers (10 μM), 10 ng of genomic DNA template, 1.0 U of Taq DNA polymerase, and water. PCR was performed based on the following amplification profile: pre-denaturation at 94°C for 5 min; 30 cycles of denaturation at 95°C for 30 s, annealing at 60°C for 30 s; extension at 72°C for 45; and final extension at 72°C for 10 min. Amplified PCR products were purified using a DNA Purification Kit (Cat No. D0033, Beyotime, Shanghai, China) and subjected to Sanger sequencing for genotype determination (Figure 1).

**Table 1:** Primer sequences used for amplification of target SNP loci

Gene	Location	SNP	Primer sequence
*IL-6* -174 G>C	7p21	rs1800795	Forward: 5′-GCCTCAATGACGACCTAAGC-3′;
			Reverse: 5′-GATTGTGCAATGTGACGTCCT-3′
*IL-8* -251 T>A	4q13-21	rs4073	Forward: 5′-CCCAAGCTTGTGTGCTCTGCTGT-3′;
			Reverse: 5′-GATTCTGCTCTTATGCCTCA-3′
*MMP-13* -77G>A	11q22	rs2252070	Forward: 5′-GAGACCCTGCTGAAACAAGAG-3′;
			Reverse: 5′-GCCAGGACCCCTGGATGCATC-3′

### Determination of IL-6, IL-8, and MMP-13 expression levels

Articular cartilage tissue (50–100 mg) was obtained from each KOA patient during artificial joint replacement surgery. Total RNA was extracted using Qiagen 74106 tissue RNA extraction kit (Qiagen, Germany). The mRNA was reverse-transcribed into cDNA using the M-MLV2 reverse transcription kit. RT-PCR was performed in 25 μl reaction volumes containing 2 μl of cDNA, 12.5 μl of 2× PCR buffer, 15 mmol/l MgCl_2_, 25 μmol/l each of the four dNTPs, 50 pmol/l primer, and 1 U of Taq enzyme. The primers used for RT-PCR were as follows: *IL-6*, 5′-GAG CTT CAG GCA GGC AGT ATC-3′ (forward) and 5′-GTA TAG ATT CTT TCC TTT GAG GC-3′ (reverse); *IL-8*, 5′-AGT GCT AAA GAA CTT AGA TG-3′ (forward) and 5′-TAT GAA TTC TCA GCC CTC TT-3′ (reverse); *MMP-13*, 5′-GGT CCC AAA CGA ACT TAA CTT ACA-3′ (forward) and 5′-CCT TGA ACG TCA TCA TCA GGA AGC-3′ (reverse); and β-actin, 5′-ACC ACC ATG GAG AAG GCT GG-3′ (forward) and 5′-CTC AGT GTA GCC CAG GAT GC-3′ (reverse). Synovial fluid (1 ml) from each KOA patient was collected and centrifuged at 14000 r/min for 20 min at 4°C, after which the supernatant was isolated for determination of IL-6 (Cat#E-EL-M0044c, Elabscience), IL-8 (Cat# E-EL-H0048c, Elabscience), and MMP-13 (Cat# E-EL-H0134c, Elabscience) levels by ELISA. All procedures were carried out in strict accordance with the manufacturers’ instructions.

### Statistical analysis

All statistical analyses were conducted using SPSS 20 software (SPSS, Inc., Chicago, IL, U.S.A.). The continuous variable was expressed as mean ± S.D., and significant differences were determined using the Student’s *t* test. Categorical variables were expressed as frequency and percentage and analyzed using the χ^2^ test. χ^2^ test was performed to analyze whether the genotype distribution was consistent with Hardy–Weinberg equilibrium. The KOA risk of *IL-6* rs1800795 (-174 G>C), *IL-8* rs4073 (-251T>A), and *MMP-13* rs2252070 (-77G>A) loci were evaluated based on the odds ratio (OR) and 95% confidence interval (CI). After adjusting for confounding factors such as age, sex, body mass index (BMI), family history, smoking, and alcohol consumption, multivariate logistic regression analysis was performed to calculate OR (95%CI). *P*<0.05 was considered statistically significant.

## Results

### Demographic characteristics

The following results were obtained based on Kellgren–Lawrence grading [14] of 400 KOA cases: 218 cases were grade 2 (54.5%), 101 cases were grade 3 (25.3%), and 81 cases were grade 4 (20.3%). We found no significant differences between the case group and the control group in terms of clinical characteristics, including age, sex, BMI, family history, smoking status, and alcohol consumption (*P*>0.05) (Table 2).

**Table 2:** Baseline data of patients in the case group and control group

Variable	Case group (*n*=400)	Control (*n*=400)	*P*-value
Age (years, mean ± S.D.)	56.6 ± 9.3	55.7±10.5	0.200
Males, number (%)	184 (46.0%)	189 (47.3%)	0.723
BMI (kg/m^2^, mean ± S.D.)	25.7 ± 1.8	25.5 ± 2.0	0.138
Family history, number (%)	40 (10.0%)	35 (8.8%)	0.544
Smoking status (*n* (%))			
Yes	164 (41.0%)	148 (37.0%)	0.383
No	236 (59.0%)	252 (63.0%)	
Alcohol consumption (*n* (%))			
Yes	172 (43.0%)	168 (42.0%)	0.775
No	228 (57.0%)	232 (58.0%)	
Kellgren–Lawrence grading			
2	218 (54.5%)	0	
3	101 (25.3%)	0	
4	81 (20.3%)	0	

### Genotype and allele frequencies of the *IL-6, IL-8*, and *MMP* SNP loci

The genotype and allele frequencies of *IL-6* rs1800795, *IL-8* rs4073, and *MMP-13* rs2252070 loci in the case and control groups are shown in Table 3. The analysis showed that the *IL-6* rs1800795, *IL-8* rs4073, and *MMP-13* rs2252070 genotypes in the control group were consistent with Hardy–Weinberg equilibrium (*P*>0.05). The recessive model of *IL-6* rs1800795 locus was associated with high risk for KOA (adjusted OR = 1.657, 95%CI = 1.396–1.866, *P*<0.001), and the rs1800795 locus C allele was a strong risk factor for KOA (Table 3). We found no significant differences in the frequencies of the *IL-8* rs4073 genotypes between the case and control groups (*P*>0.05). The dominant and recessive models of *IL-8* rs4073 showed no significant difference in KOA risk (*P*>0.05) (Table 3). The dominant and recessive models of the *MMP-13* rs2252070 were found to be associated with high risk for KOA (dominant model: adjusted OR = 1.271, 95%CI = 1.095–1.480, *P*=0.001; recessive model: adjusted OR = 1.361, 95% CI = 1.151–1.569, *P*<0.001) (Table 3). The A allele in the *MMP-13* rs2252070 locus was found to be a strong risk factor for KOA (*P*<0.001).

**Table 3:** Genotype and allele frequencies of SNP loci in the case and control groups

Variable	Case group (*n*=400)	Control (*n*=400)	Crude OR (95%CI)	*P*-value	Adjusted OR (95%CI)	*P*-value
IL-6 rs1800795						
Genotype						
GG	258 (64.5%)	278 (69.5%)	1.00			
GC	82 (20.5%)	105 (26.3%)	0.841 (0.594–1.193)	0.312	0.911 (0.746–1.096)	0.355
CC	60 (15.0%)	17 (4.3%)	3.803 (2.098–6.968)	<0.001	1.619 (1.358–1.832)	<0.001
Dominant model						
GG	258 (64.5%)	278 (69.5%)	1.00			
GC+CC	142 (35.5%)	122 (30.5%)	1.254 (0.923–1.704)	0.133	1.117 (0.961–1.290)	0.153
Recessive model						
GG+GC	340 (85.0%)	383 (95.8%)	1.00			
CC	60 (15.0%)	17 (4.3%)	3.976 (2.210–7.230)	<0.001	1.657 (1.396–1.866)	<0.001
Alleles						
G	598 (74.8%)	661 (82.6%)	1.00			
C	202 (25.3%)	139 (17.4%)	1.606 (1.251–2.063)	<0.001	1.247 (1.115–1.383)	<0.001
IL-8 rs4073						
Genotype						
TT	101 (25.3%)	117 (29.3%)	1.00			
TA	189 (47.3%)	186 (46.5%)	1.177 (0.831–1.668)	0.339	1.088 (0.911–1.310)	0.384
AA	110 (27.5%)	97 (24.3%)	1.314 (0.881–1.959)	0.160	1.147 (0.938–1.401)	0.191
Dominant model						
TT	101 (25.3%)	117 (29.3%)	1.00			
TA+AA	299 (74.7%)	283 (70.7%)	1.224 (0.885–1.692)	0.204	1.109 (0.942–1.322)	0.234
Recessive model						
TT+TA	290 (72.5%)	303 (75.8%)	1.00			
AA	110 (27.5%)	97 (24.3%)	1.185 (0.852–1.647)	0.294	1.087 (0.922–1.264)	0.333
Alleles						
T	391 (48.9%)	420 (52.5%)	1.00			
A	409 (51.1%)	380 (47.5%)	1.156 (0.946–1.414)	0.147	1.075 (0.972–1.189)	0.161
MMP-13 rs2252070						
Genotype						
GG	149 (37.3%)	195 (48.8%)	1.00			
GA	165 (41.3%)	157 (39.3%)	1.375 (1.002–1.889)	0.041	1.183 (1.001–1.398)	0.049
AA	86 (21.5%)	48 (12.0%)	2.345 (1.521–3.620)	<0.001	1.482 (1.226–1.754)	<0.001
Dominant model						
GG	149 (37.3%)	195 (48.8%)	1.00			
GA+AA	251 (62.8%)	205 (51.3%)	1.602 (1.197–2.146)	<0.001	1.271 (1.095–1.480)	0.001
Recessive model						
GG+GA	314 (78.5%)	352 (88.0%)	1.00			
AA	86 (21.5%)	48 (12.0%)	2.008 (1.344–3.005)	<0.001	1.361 (1.151–1.569)	<0.001
Alleles						
G	463 (57.9%)	547 (68.4%)	1.00			
A	337 (42.1%)	253 (31.6%)	1.574 (1.276–1.942)	<0.001	1.246 (1.127–1.373)	<0.001

### Correlation between *IL-6, IL-8*, and *MMP* SNPs and KOA disease severity

Kellgren–Lawrence grading of KOA patients harboring the *IL-6* rs1800795, *IL-8* rs4073, and *MMP-13* rs2252070 genotypes is shown in Table 4. The analysis showed that the *IL-6* rs1800795 SNP was associated with higher KOA disease severity (*P*<0.001). The G>C mutation was associated with high KOA disease severity. The *IL-8* rs4073 locus SNP showed no correlation with KOA severity (*P*=0.186). The G>A mutation at *MMP-13* rs2252070 locus was positively correlated with KOA severity (*P*<0.001).

**Table 4:** Correlation between *IL-6, IL-8*, and *MMP-13* SNPs and Kellgren–Lawrence grading in KOA patients

Variable	2 (*n*=218)	3 (*n*=101)	4 (*n*=81)	*P*-value
IL-6 rs1800795				
GG (*n*=258)	153 (70.2%)	81 (80.2%)	24 (29.6%)	<0.001
GC (*n*=82)	48 (22.0%)	14 (13.9%)	20 (24.7%)	
CC (*n*=60)	17 (7.8%)	6 (5.9%)	37 (45.7%)	
IL-8 rs4073				
TT (*n*=101)	64 (24.8%)	21 (8.1%)	16 (6.2%)	0.186
TA (*n*=189)	98 (38.0%)	54 (20.9%)	37 (14.3%)	
AA (*n*=110)	56 (21.7%)	26 (10.1%)	28 (10.9%)	
MMP-13 rs2252070				
GG (*n*=149)	110 (42.6%)	28 (10.9%)	11 (4.3%)	<0.001
GA (*n*=165)	102 (39.5%)	39 (15.1%)	24 (9.3%)	
AA (*n*=86)	6 (2.3%)	34 (13.2%)	46 (17.8%)	

### Relationship between *IL-6, IL-8*, and *MMP-13* SNPs and mRNA expression

**Figure 2:**
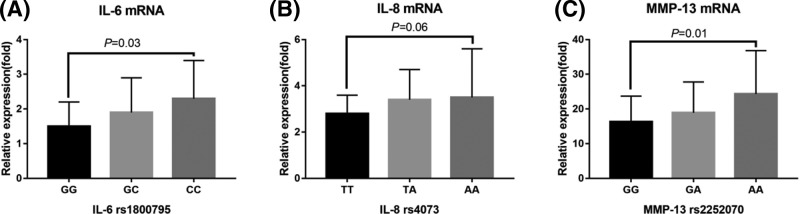
Correlation between SNPs in the *IL-6, IL-8*, and *MMP-13* genes and mRNA expression levels (**A**) Comparison of *IL-6* mRNA expression levels in osteoblasts of KOA patients with the different *IL-6* rs1800795 genotypes. (**B**) Comparison of *IL-8* mRNA expression in osteoblasts of KOA patients with the different *IL-8* rs4073 genotypes. (**C**) Comparison of *MMP-13* mRNA expression levels and genotypes of *MMP-13* rs2252070 locus in the osteoblasts of KOA patients.

Patients harboring the G>A mutation at the *IL-6* rs1800795 locus showed significantly higher *IL-6* mRNA expression levels (*P*=0.03) (Figure 2A). There were no significant differences in *IL-8* mRNA expression levels in osteoblasts from KOA patients with different genotypes at the *IL-8* rs4073 locus (*P*=0.06) (Figure 2B). *MMP-13* mRNA expression levels were up-regulated in the osteoblasts of individuals harboring the wild-type, heterozygote, and mutant homozygote genotypes in the *MMP-13* rs2252070 locus (*P*=0.01) (Figure 2C).

### Correlation between SNPs in the *IL-6, IL-8*, and *MMP-13* genes and their mRNA expression

**Figure 3:**
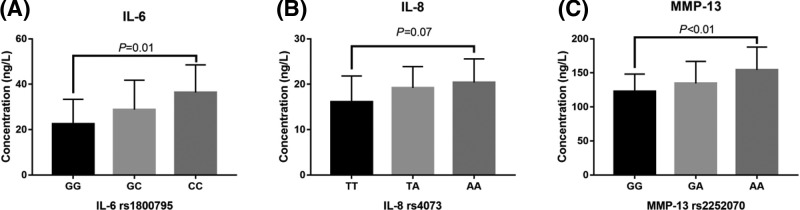
Correlation between SNPs in the *IL-6, IL-8*, and *MMP-13* genes and mRNA expression levels (**A**) Comparison of IL-6 expression levels in the synovial fluids of KOA patients harboring the different *IL-6* rs1800795 genotypes. (**B**) IL-8 expression levels in the synovial fluids of KOA patients harboring the different *IL-8* rs4073 genotypes. (**C**) MMP-13 expression levels in the synovial fluids of KOA patients harboring the different *MMP-13* rs2252070 genotypes.

KOA patients of harboring the IL-6 rs1800795 loci G>A mutation showed significantly higher IL-6 levels in the synovial fluid (*P*=0.01) (Figure 3A). We found no statistically significant difference in IL-8 levels in the synovial fluids of KOA patients harboring different genotypes in the *IL-8* rs4073 locus (*P*=0.07) (Figure 3B). The G>A mutation in MMP-13 rs2252070 was associated with higher MMP-13 levels in the synovial fluids of KOA patients (*P*<0.01) (Figure 3C).

## Discussion

The present study analyzed the relationship between the SNPs in the *IL-6* rs1800795 (-174 G/C), the *IL-8* rs4073 (-251A/T), the *MMP-13* rs2252070 (-77A/G) loci and KOA susceptibility and disease severity. Our findings showed that the *IL-8* rs4073 (-251T>A) mutation was not associated with KOA susceptibility and disease severity. The *IL-6* rs1800795 (-174 G>C) and the *MMP-13* rs2252070 (-77G>A) mutations were found to be associated with KOA susceptibility and disease severity. In addition, patients harboring the *IL-6* rs1800795 (-174 G>C) and the *MMP-13* rs2252070 (-77G>A) mutations showed up-regulated expression of the corresponding inflammatory cytokines [15–17].

Accumulating evidence has shown that OA occurrence is caused by various factors. The OA is characterized by pathological changes in the cartilage, subchondral bone, ligaments, synovium, joint capsules, and surrounding joint tissues, all of which participate in the pathogenesis of OA [18]. During the progression of OA, different risk factors may ultimately cause OA through a common pathway that primarily affects the synovial fluid and subchondral bone [19]. Osteoblasts are critical for maintaining the subchondral bone, and the biological phenotype of the osteoblasts determines subchondral bone remodeling, bone sclerosis, and reduced bone mineralization capacity [20]. A previous study by Sakao et al. [9] showed that osteoblasts in the osteophytes of OA patients can produce higher levels of IL-6, IL-8, and MMP-13 than the subchondral bone osteoblasts of healthy individuals. These inflammatory factors participate in subchondral bone remodeling, angiogenesis, and expression of inflammatory proteins via related signaling pathways, thereby influencing the occurrence and progression of OA. The expression of IL-6, IL-8, and MMP-13 were significantly correlated with gene polymorphism. The present study analyzed the relationship between the SNPs of these three genes and KOA.

The *IL-6* gene is located on chromosome 7p21. IL-6 is a multifunctional cytokine that can act as an inflammatory mediator and a regulator of endocrine and metabolic functions. Sun et al. [10] showed that the rs1800795 (-174 G>C) in the *IL-6* gene is associated with coronary artery disease (CAD). Both the dominant and recessive CAD is increasing. However, our findings showed that rs1800795 (-174 G>C) increases KOA risk only in the recessive model. These results could be attributed to deviations of the rs1800795 locus from the Hardy–Weinberg equilibrium in the control population in Sun et al. study (*P*<0.001) [10]. Their study population was from Jilin, and its secondary allele frequency (MAF:C) of the rs1800795 locus was 18.20%, while the data from the 1000 Genomes database showed that the MAF(C) was 9.02% in the U.S. population. MAF(C) was as high as 51.52% in the Western European population, indicating that the SNP locus was significantly associated with ethnic differences. Our current results indicated that the *IL-6* rs1800795 (-174 G>C) mutation is not only related to higher KOA risk and higher disease severity, but also to higher severity of arthritis. We hypothesized that the observed correlation was caused by dramatic up-regulation of IL-6 levels. Furthermore, mRNA expression levels of IL-6 in the osteoblasts of mutant KOA patients were considerably higher than those in the osteoblasts of individuals harboring the wild-type genotype. Furthermore, individuals harboring the -174G>C mutation showed higher IL-6 levels in the synovial fluid, leading to disease progression of KOA.

The *IL-8* gene is located in the human chromosome 4q13-21. The *IL-8* rs4073 (-251T>A) site is located in the transcription initiation region and is potentially involved in the regulation of IL-8 expression. Previous studies showed that the *IL-8* rs4073 (-251T>A) polymorphism is associated with the occurrence of various diseases [21,22]. Andia et al. [23] showed that the carriers of allele A at IL-8 rs4073 locus had a high level of IL-8 expression, which was consistent with the results of this study. According to data from 1000 Genomes database, the frequency of allele A at IL8 rs4073 locus was 42.38% in Southern Chinese Han population, 85.35% in Esan Nigeria, and 81.97% in Americans. The frequency of allele A in the present study was 47.5%, which was close to the results in the database, indicating that the selection of samples in the present study was representative. However, our current results showed no significant association between *IL-8* rs4073 (-251T>A) polymorphisms and the occurrence and disease severity of KOA. We found no significant differences in *IL-8* expression levels amongst KOA patients harboring the *IL-8* rs4073 (-251T>A) genotype. Considering that IL-8 can be secreted by various cell types, a compensatory expression mechanism is potentially responsible for maintaining stable IL-8 expression levels.

The *MMP-13* gene is located on the human chromosome 11q22, and the rs2252070 (-77G>A) polymorphism in the *MMP-13* promoter region is associated with the development of various diseases [17,24]. The rs2252070 (-77G>A) polymorphism in the *MMP-13* promoter region is known to influence MMP-13 transcription. The mutant A allele is associated with two-fold higher transcriptional activity of the *MMP-13* gene compared with that of the G allele [25]. According to data from 1000 Genomes database, the G allele frequency of MMP-13 rs2252070 (-77G > A) locus was 44.76%, and the A allele frequency was 55.24%. In Americans of Africa, G allele frequency was 33.61%, A allele frequency was 66.39%. The G allele frequency was 57.21% and A allele frequency was 42.79% in Japanese in Tokyo. Therefore, the allele frequencies of MMP-13 rs2252070 locus were significantly different amongst different races. In the present study, the G allele frequency was 68.4%, which was close to that of Americans of Africa. The analysis may be related to the small sample size in the present study. Our findings revealed that both the dominant and recessive models of the *MMP-13* rs2252070 locus were associated with higher KOA risk. Furthermore, individuals harboring the mutant rs2252070 genotype showed higher *MMP-13* mRNA expression levels in the osteoblasts and the synovial fluids of KOA patients were higher than those in the osteoblasts and synovial fluids of the wild-type individuals, indicating that the -77G>A mutation increases MMP-13 transcriptional activity and up-regulates MMP-13 expression.

Our current results showed that the rs1800795 (-174 G>C) and rs2252070 (-77G>A) polymorphisms in the promoter region of the *IL-6* gene were associated with the occurrence and progression of KOA. However, KOA occurrence and progression are influenced by multiple factors. Subsequent studies should investigate the influence of other genes, the environment, and gene–gene interactions to elucidate the mechanisms underlying the occurrence and progression of KOA.

Our current study has several limitations. First, considering the difficulty of sample collection of osteoblasts and synovial fluids, polymorphism data on the promoter regions of the *IL-6* rs1800795 (-174G>C) and the *MMP-13* rs2252070 (-77G>A) loci and the expression levels of the corresponding cytokines were not obtained. In addition, the contribution of external mechanical forces on the development and progression of knee arthritis could not be ruled out.

## Conclusion

Our findings showed that *IL-8* rs4073 (-251T>A) mutation was not associated with KOA susceptibility. The *IL-6* rs1800795 (-174 G>C) and *MMP-13* rs2252070 (-77G>A) mutations are associated with higher KOA susceptibility and disease severity and contribute to KOA pathogenesis via up-regulation of IL-6 and MMP-13 expression levels.

## Data availability statement

All data generated and/or analyzed during the current study are available from the corresponding author upon reasonable request.

## Abbreviations

BMI: body mass index
CAD: coronary artery disease
CI: confidence interval
IL: interleukin
KOA: knee osteoarthritis
MMP-13: matrix metalloproteinase-13
OA: osteoarthritis
OR: odds ratio
SNP: single nucleotide polymorphism
